# The impact of community of inquiry and self-efficacy on student attitudes in sustained remote health professions learning environments

**DOI:** 10.1186/s12909-023-04382-2

**Published:** 2023-06-28

**Authors:** Amanda K. Burbage, Yuane Jia, Thuha Hoang

**Affiliations:** 1 Eastern Virginia Medical School, School of Health Professions, P.O. Box 1980, Norfolk, VA 23501-1980 USA; 2 School of Health Professions, The State University of New Jersey, Newark, NJ USA; 3 Department of Physical Therapy, Louisiana State University Health Sciences Center- New Orleans, New Orleans, LA USA

**Keywords:** Community of inquiry, COVID, Mediation model, Presence, Self-efficacy, Sustained remote learning

## Abstract

**Background:**

Sustained remote learning environments, like those experienced in late 2020 due to the COVID-19 pandemic, share characteristics with online courses but were not intentionally designed to delivered virtually. The purpose of this study was to investigate the impact of Community of Inquiry, a widely used online learning environment framework, and self-efficacy on perceived student attitudes within sustained remote learning environments.

**Methods:**

An interinstitutional team of health professions education researchers collected survey data from 205 students representing a wide range of health professions in five U.S. institutions. Latent mediation models under structural equation modeling framework were used to examine whether student self-efficacy mediates the relationship between Community of Inquiry presence and student’s favorability of sustained remote learning delivered in the prolonged stages of the COVID-19 pandemic.

**Results:**

Higher levels of teaching presence and social presence in the remote learning environment were associated with higher levels of remote learning self-efficacy which, in turn, predicts variance in positive attitudes toward remote learning. When mediated by self-efficacy, significant variance in student’s favorability of sustained remote learning was explained by teaching presence (61%), social presence (64%), and cognitive presence (88%) and self-efficacy. Significant direct and indirect effects for teaching and social presence, and only direct effects for cognitive presence were observed.

**Conclusions:**

This study establishes the Community of Inquiry and its three presence types as a relevant and stable framework for investigating sustained remote health professions teaching and learning environments, not only carefully designed online learning environments. Faculty may focus course design strategies which enhance presence and increase student self-efficacy for the sustained remote learning environment.

In March 2020 COVID-19 forced students and faculty into lockdown and remote learning conditions for approximately three months [1, 2]. During this emergency remote transition (ERT), the opportunity to carefully select course modality informed by self-efficacy for a particular learning environment, and the time required to design a course which effectively facilitates online learning [3] was not available. By September 2020, higher education institutions faced decisions about returning to campus, and many institutions continued with virtual learning [4, 5]. At this stage of the pandemic, students and faculty emerged from rapidly ERT courses into sustained remote learning environments (SRLEs) [6] while the return to face-to-face instruction was intermittent. SRLEs can be characterized as sharing more characteristics with online courses because planning is more feasible, and the participants have gained distance learning experiences, but SRLEs are not equivalent to online learning environments because pedagogical strategies for instruction, engagement, and assessment were not intentionally used in advance of course design or delivery [6, 7].

Community of Inquiry (CoI) [8] is widely used to situate practices of online learning and establish elements of learning experiences related to student attitudes and outcomes [9]. Researchers have primarily used CoI to investigate contexts in which students chose online modalities and faculty intentionally designed online courses. Additional factors enriching CoI include student attributes, such as self-efficacy. Self-efficacy has been applied to CoI investigations [10], related to perceived attitudes and outcomes [11], and connected to future intentions for online learning [12].

In evolving and challenging conditions such as the COVID-19 pandemic, understanding the role of self-efficacy in SRLEs may be the key to the student’s level of engagement and learning outcomes [13, 14]. Studies of self-efficacy and engagement during the early stages of the pandemic suggested that higher levels of self-efficacy enhanced student’s participation and attitude towards online learning [15, 16]. However, investigations of self-efficacy during COVID-19 have primarily used alternative theories (e.g. social cognitive theory, demands-resources-theory) to frame investigations, and have narrowed data collection and analysis to the ERT context, ignoring the SRLE context.

In summary, adapting courses to new modalities of learning require social, pedagogical, managerial, and technical faculty skillsets [17, 18] which develop with training and practice, little of which was available during the COVID-19 sustained remote learning stage [19]. Because institutions must remain ready for another learning modality-altering situation [20], a better understanding of CoI and self-efficacy in SRLE may help address gaps in current practices and outcomes.

## Community of inquiry

Using social-constructivist theory, the CoI framework was built on three dimensions: teaching presence (design and facilitation of the course), social presence (to authentically project oneself online), and cognitive presence (creating and connecting meaningful ideas) [21]. Although each presence is unique, having all three presence-types in a course fosters enhanced learning experiences for students [22].

The cognitive, social, and teaching presence constructs of CoI are typically measured using a 34-item Likert-scale instrument. Authors have demonstrated temporal stability and contextual validity of the relationship of the three presences [23–25]. CoI has been used with online [26], blended courses [27], undergraduate, and graduate [28] level learners. CoI is relevant to international educational contexts [29, 30] and the instrument has been validated in multiple languages [31–33].

Implementation of CoI presence types relies on course design strategies that require planning [34, 35]. Instructors may include topic self-selection, role play, or reflective practices to foster cognitive presence [36]. Social presence may be included in course design through technical support, promotion of informal relationships, use of profiles and photos, and activities that draw out student feelings and experiences [37]. Finally, an instructor creates teaching presence through narrative, facilitating discourse, and detailed feedback [38]. However, the required “design and organization” ([35], p. 6) typical of online courses was not present in courses that rapidly transitioned, and then persisted, in the SRLE. Thus, questions about CoI stability in SRLE remain.

### Self-efficacy

Self-efficacy considers students’ beliefs about their skills and abilities [39]. Within the context of this study, self-efficacy for learning in SRLE is highly relevant as course modalities continued to fluctuate throughout the COVID-19 pandemic [40, 41].

Regarding CoI in online courses, teaching presence positively predicted self-efficacy, and self-efficacy mediated the effect between social and cognitive presence [42]. Martin et al. [9] encouraged scholars to investigate student attributes like self-efficacy alongside CoI to better explain factors which may mediate or moderate component relationships. Moreover, self-efficacy may be lower for marginalized populations such as females in STEM [43] and non-traditional students [44], thus making it a vital consideration for equity.

### Attitudes towards learning

Understanding student attitudes is useful within the context of self-efficacy and SRLE. Chu et al. [45] found perceived outcomes and student satisfaction could be improved through facilitation of student interactions and course design fostering self-direction. Faculty that prepare students to learn in mediated environments, prioritize effective online systems use, and focus on practical problems helped improve student attitudes toward learning [45].

Attitudes towards learning have been investigated in face-to-face and online environments, consistently indicating a positive relationship to performance [46–48]. Attitudes regarding motivation toward learning are linked to achievement [49] and satisfaction [50] in health professions. Favorability and satisfaction as a perceived health professions student outcome warrants investigation, not only for its positive links to student attitudes and outcomes [51] but also for its accessibility and practical implications for faculty and course designers [52], particularly those considering CoI strategies to improve SRLEs.

CoI presence is a clear positive predictor of student attitudes in online contexts. Richardson et al. [37] conducted a meta-analysis of social presence, finding COI explained student satisfaction, and that the relationship was moderated by course length where longer courses showed stronger social presence relationships. Cognitive presence predicted satisfaction, persistence, and learning flow [53, 54]. Khalid et al. ([55], p. 66) summarized the relationship between teaching presence and satisfaction as reciprocal, and “the construct of teaching presence in the CoI framework is vital in sustaining course satisfaction”.

Lockdowns continue to occur globally [56, 57] and may continue to be used to prevent infection spread [58]. Therefore, the purpose of this study is to gain a better understanding of CoI in SRLEs. Increased understanding is particularly necessary in health professions which require the delivery of practical courses such as anatomy, clinical care, and ultrasound techniques and are uniquely impacted by accelerated technological transitions [59, 60]. In essence, applying the well supported CoI lens on the emerging SRLE modality represents both a theoretical and practical contribution to the field.

The following research questions were addressed:

- RQ_1_: What is the relationship of Community of Inquiry presence types and favorability of sustained remote learning environments?
- RQ_2_: How does self-efficacy mediate the relationship between Community of Inquiry presence types and favorability of sustained remote learning environments?

## Methods

An interinstitutional team of health professions education (HPE) researchers from six U.S. universities and academic health centers collaborated to validate a revised CoI instrument. Following a comprehensive literature review of student online learning, the research team identified important constructs and corresponding items. Then, researchers revised and ranked the items to ensure fit for measuring modality change and construct. The survey included a series of demographic questions followed by the 29 items statements on a six-point Likert scale from ‘strongly disagree’ (1) to ‘strongly agree’ (6). Each item asked students to rate their agreement with statements about experiences with learning and instruction during the global pandemic. The measurement constructs included self-efficacy, attitudes towards remote learning online, teaching presence, social presence, and cognitive presence. The 29-item instrument was found to have strong construct validity [61].

The overall cross-sectional study design analyzed self-reported data collected in the CoI instrument [61]. The questionnaire and methodology for this study was first approved by Louisiana State University Health Sciences Center University Institutional Review Board, then subsequently approved by the boards at participating institutions. Researchers distributed the survey in the fall 2021 to deans and program directors of health professions education programs at their respective institutions. Equal opportunity to participate in the survey was provided at each institution, limiting selection bias. The recruitment email contained a web-based link to an online consent document and the 29 survey items. Data were collected from 205 students enrolled in health professions curriculums, 11 students who answered less than half of the survey were dropped from the analyses, leaving the final analytic sample of 194 students, primarily representing 5 institutions.

### Participants

A representative convenience sample of 194 students participated in the study. Majority of the students were White (63%) and females (74%). Most students were under the age of 35 years (82%), and smaller portions were between 35 and 44 (10%), or 45 and older (8%). The students were enrolled in their health professions programs at their institutions from fall 2019 to the fall 2021. The sample consisted of students from a wide range of programs with the majority in Nursing (18.5%), Doctor of Medicine (MD) (17.6%), and Physician Assistant (12.2%) programs. Forty six percent of the students were in doctorate degree, 33% were in master’s, and 21% were in bachelor’s or certificate/associate degree. Seventy five percent of students had online course experience prior to the pandemic; however, only 22% indicated “quite a bit” or “a great deal” of experience with online learning before the pandemic.

### Measures

#### Learning modality change self-efficacy scale

To assess students’ perceived self-efficacy in the learning modality change during the pandemic, a common stem introduced survey items: “After experiencing a change in course delivery/learning modality as a result of the COVID-19 pandemic… I feel confident in…”. The revised 11 item scale [61] based on two existing self-efficacy scales in the literature, has been validated in a confirmatory factor analysis in a similar student sample. The overall internal reliability was 0.95. The scale has three subscales where 3 items measure online learning task self-efficacy, 4 items measure instructor and peer interaction and communication self-efficacy, and 4 items measure self-regulation and motivation efficacy. The scale score of self-efficacy was calculated by averaging all 11 items. The internal reliability for each of the three subscales ranged from 0.78 to 0.92. Exemplary items are: I feel confident in taking an online quiz/test, I can manage study time for my online courses by setting goals.

#### Learning modality change coI scale

A revised 14-item scale (4 items on cognitive presence, 5 items on social presence, and 5 items on teaching presence) was validated in a confirmatory factor analysis in a similar student sample [61], showing good data-model fit. The research team modified the survey items to reflect the changes in the perceived impacts of remote learning with traditional face-to-face classes. A common stem introduced the items for the CoI portion of the survey, which read as follows: "After my courses went online due to the pandemic…". The exemplary items are: The instructors were able to guide the class effectively to completing the course activities; I was equally involved in interactions with peers as I was in face-to-face courses. The overall Cronbach’s alpha reliability coefficients were estimated for the new Learning Modality Change Community of Inquiry scale was 0.92. The internal reliability for each of the three subscales of Learning Modality Change CoI scale ranged from 0.89 to 0.92. Subscale scores of CoI were calculated by averaging the respective items in each subscale.

#### Favorability of sustained remote learning

To assess students’ Favorability of Sustained Remote Learning (FSRL), two items were adapted from a validated study measuring attitudes change towards online learning [45], another two items developed by the research team were also included in the survey. The four items were as such: As a result of taking online courses during the pandemic…I prefer online classes to face to face classes, I believe that online classes could replace face to face classes, I am more willing to enroll in online classes than I was before, and I discovered that online learning is not for me. A six-point Likert scale was used. The internal reliability was 0.92. The scale score of FSRL was calculated averaging all 4 items.

### Statistical analysis

First, descriptive analysis was conducted to gain information about the subjects and variation in participant characteristics. Second, inferential analysis isolated specific effects of each CoI presence type, as mediated by self-efficacy, on favorability of SRLEs. Latent mediation models and Maximum likelihood estimation with robust standard errors (MLR) were used with structural equation modeling analysis. MLR is robust to no-normal data, and it can handle missing information [62]. Additionally, simulation studies demonstrated that MLR and categorical least squares produce similar results even when ordinal variables of six to seven categories were used [63]. To account for potentially non-normal distribution of the indirect effect and to address concerns of statistical power [64] a non-symmetric and bias-corrected bootstrap confidence interval was requested in Mplus.

Since students nested within institutions, the intraclass correlation coefficient of outcome variable FSRL (ICC = 0.04) was computed to consider whether there is evidence of clustered observations within institutions. Heck et al. [65] suggested 0.05 as a rough cutoff of substantial clustering. Other researchers indicated that even trivial amounts of clustering may still have substantial effects on inferences [66]. As a robustness check, to account for the nested nature of data (i.e., students nested within institutions), dummy coded variables with institutions were added as covariates to the mediation models.

## Results

Descriptive statistics for observed scale scores are presented in Table 1. Each of the scale scores was averaged by its number of items so that all the scale score means are comparable against the scale of 1 to 6. The overall mean for students’ rating about the favorability of sustained remote learning was 3.44 with a standard deviation of 1.53, indicating moderate attitudes toward sustained remote learning from health professions students. Social presence was found to be the lowest (*M* = 2.72 out of 6) among health professions students, followed by cognitive presence and teaching presence. By contrast, students reported a high level of self-efficacy (*M* = 4.23) with sustained remote learning.

**Table 1:** Descriptive of observed scale scores

Scale	min	max	*M*	*SD*
Self-Efficacy	2	6	4.23	0.97
Favorability of Sustained Remote Learning	1	6	3.44	1.53
Teaching Presence	1	6	3.55	1.23
Social Presence	1	6	2.72	1.36
Cognitive Presence	1	6	3.02	1.34

Bivariate correlations between observed scale scores are shown in Table 2. The correlations between observed scale scores of three CoI components and FSRL ranged from 0.64 to 0.85, self-efficacy was associated with all three CoI components, ranging from 0.66 to 0.71. Finally, FSRL was associated with self-efficacy (*r* = 0.70). All the correlations are statistically significant (*p* < 0.001).

**Table 2:** Bivariate correlations between observed scale scores

	Self-Efficacy	Favorability of SRL	Teaching Presence	Social Presence	Cognitive Presence
Self-Efficacy	1.00				
Favorability of Sustained Remote Learning	0.70	1.00			
Teaching Presence	0.71	0.64	1.00		
Social Presence	0.66	0.65	0.67	1.00	
Cognitive Presence	0.70	0.85	0.65	0.78	1.00

*Note*. All correlations are statistically significant, all *p* values < .001

**Fig. 1:**
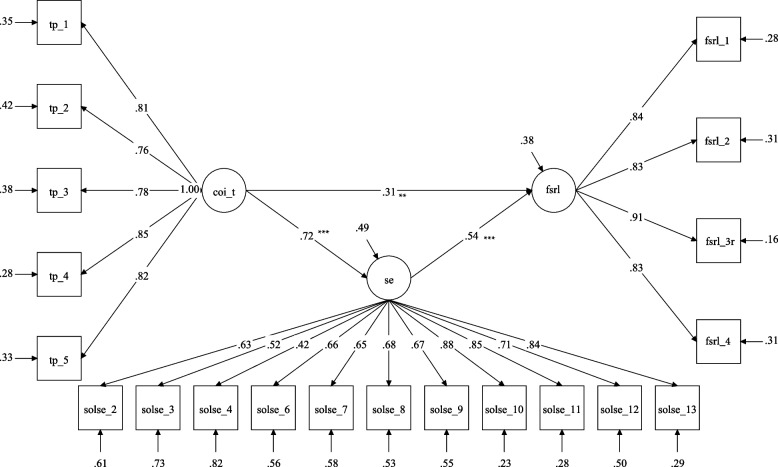
Teaching Presence and Self-Efficacy Mediation Model*.* Note: TP = Teaching Presence, CoI_T = CoI-Teaching Presence, SOLSE = Student perceived online learning self-efficacy, FSRL = Favorability of Sustained Remote Learning; Squares represent observed item scores, circles represent underlying latent construct measured indirectly through those observed items. The latent constructs remove the measurement error in the observed scores, which leads to more accurate estimates of the relationship between latent constructs. This figure demonstrates significant direct effect between teaching presence and favorability of sustained remote learning and significant indirect effect through self-efficacy. Thus, higher levels of presence, and higher levels of self-efficacy, can predict positive attitudes toward sustained remote learning environments.* *p* < .05. ** *p *< .01. *** *p* < .001

**Fig. 2:**
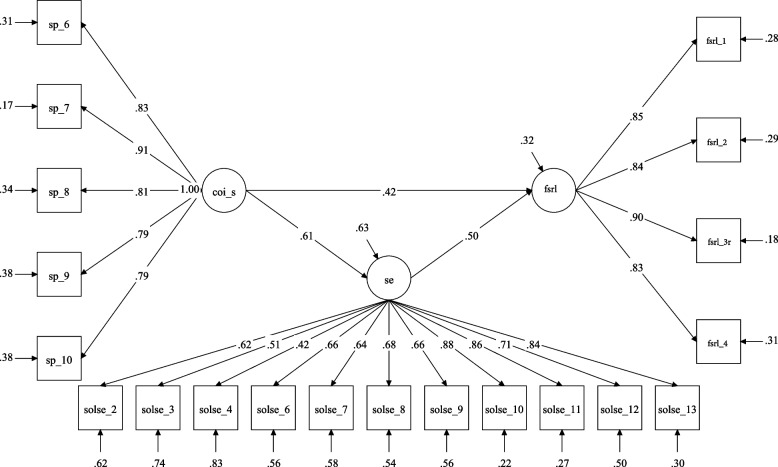
Social Presence and Self-Efficacy Mediation Model*.* Note: SP = Social Presence, CoI_S = CoI-Social Presence, SOLSE = Student perceived online learning self-efficacy, FSRL = Favorability of Sustained Remote Learning; Squares represent observed item scores, circles represent underlying latent construct measured indirectly through those observed items. The latent constructs remove the measurement error in the observed scores, which leads to more accurate estimates of the relationship between latent constructs. This figure demonstrates significant direct effect between social presence and favorability of sustained remote learning and significant indirect effect through self-efficacy. Thus, higher levels of presence, and higher levels of self-efficacy, can predict positive attitudes toward sustained remote learning environments.* *p* < .05, ** *p* < .01, *** *p* < .001

Standardized and unstandardized model coefficients and indirect effects are shown in Table 3 for three latent mediation models. Model fit indices indicated acceptable model fit with Comparative Fit Index (CFI)/ Tucker‐Lewis Index (TLI) greater than 0.9, and the Root Mean Square Error of Approximation (RMSEA)/Standardized Root Mean Square Residual (SRMR) less than 0.8 [67]. In model 1, teaching presence was found to be directly (β_standardized_ = 0.26, *p* < 0.01) and indirectly (indirect effect = 0.53, *p* < 0.05, 95% CI = [0.36, 0.74]) associated with the FSRL through self-efficacy. See Fig. 1 for more detail of the model. In model 2, social presence was found to be directly (β_standardized_ = 0.35, *p* < 0.01) and indirectly (indirect effect = 0.33, *p* < 0.05, 95% CI = [0.22, 0.47]) associated with the FSRL through self-efficacy (see Fig. 2). The significant, positive indirect effects indicated that students perceived higher levels of teaching presence and social presence in the remote learning environment were associated with higher levels of remote learning self-efficacy which, in turn, predicts variance in positive attitudes toward remote learning.

**Table 3:** Latent mediation model estimation results

		β_standardized_	β_unstandardized_	95% CI			
**Model 1**							
FSRL on	SE	0.54	1.22	[0.76, 1.73]	***		
	COI_T	0.31	0.42	[0.18, 0.70]	**		
SE on	COI_T	0.72	0.43	[0.30, 0.59]	***		
Indirect Effect		–	0.53	[0.36, 0.74]	*		
R squared for FSRL						0.62	
**Model 2**							
FSRL on	SE	0.50	1.15	[0.77, 1.57]	***		
	COI_S	0.42	0.46	[0.30, 0.63]	***		
SE on	COI_S	0.61	0.29	[0.20, 0.38]	***		
Indirect Effect		–	0.33	[0.22, 0.47]	*		
R squared for FSRL						0.68	
**Model 3**							
FSRL on	SE	0.15	0.35	[-0.02, 0.69]	#		
	COI_C	0.82	1.00	[0.80, 1.24]	***		
SE on	COI_C	0.73	0.38	[0.28, 0.52]	***		
Indirect Effect		–	0.13	[-0.004, 0.26]	#		
R squared for FSRL						0.88	
	**Chi-square**	**df**	**CFI**	**TLI**	**RMSEA**	**SRMR**	
**Model 1**	351.01	162	0.92	0.91	0.08	0.07	
**Model 2**	362.46	161	0.92	0.91	0.08	0.08	
**Model 3**	325.26	144	0.93	0.91	0.08	0.06	

*Note.* FSRL = Favorability of Sustained Remote Learning, SE = Self-Efficacy, COI_T = Teaching Presence, COI_S = Social Presence, COI_C = Cognitive Presence

^*^ *p* < .05. ** *p* < .01. *** *p* < .001. # *p* < .10

**Fig. 3:**
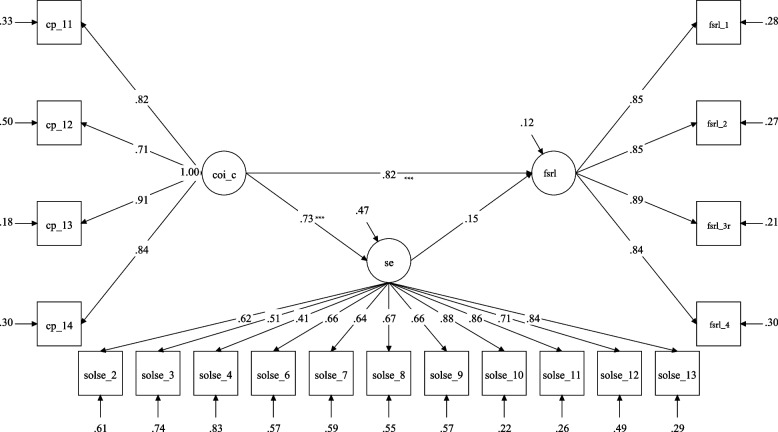
Cognitive Presence and Self-Efficacy Mediation Model. Note: CP = Cognitive Presence, CoI_C = CoI-Cognitive Presence, SOLSE = Student perceived online learning self-efficacy, FSRL = Favorability of Sustained Remote Learning; Squares represent observed item scores, circles represent underlying latent construct measured indirectly through those observed items. The latent constructs remove the measurement error in the observed scores, which leads to more accurate estimates of the relationship between latent constructs. This figure demonstrates significant direct effect between teaching presence and favorability of sustained remote learning. Thus, higher levels of presence can predict positive attitudes toward sustained remote learning environments. The indirect role of self-efficacy was not a significant mediator of attitudes toward sustained remote learning environments.* *p* < .05, ** *p* < .01, *** *p* < .001

As shown in Fig. 3, cognitive presence was found to be directly (β_standardized_ = 0.83, *p* < 0.01) associated with the FSRL in model 3. However, the indirect effect through self-efficacy was not found to be significant (indirect effect = 0.13, *p* > 0.05, 95% CI = [-0.004, 0.26], *ns*) in this sample. Notably, the bias-corrected 90% CI = [0.03, 0.24] did not include zero, showing a significant indirect effect at *p* < 0.10.

In summary, each of the Community of Inquiry factors, that is, teaching presence and self-efficacy, social presence and self-efficacy, and cognitive presence and self-efficacy (although not showing a significant indirect effect) explained 62%, 68%, and 88% of the variance respectively in favorability of sustained remote learning. As a robustness check against nested samples (students nested within institutions), 5 dummy coded institution variables were added as covariates to the mediation models [68]. With all else remaining unchanged, adding dummy coded variables did not substantially change the model results.

## Discussion

Several significant results emanate from this study. In our model analysis all CoI presence types explained significant portions of variance in FSRL, but only teaching presence and social presence demonstrated significant mediation effects through self-efficacy. The initial review of the factor loadings on the latent constructs were high and significant suggesting strong construct validity [61] and, therefore, justification to extend CoI as a framework for this novel learning environment. Further, FSRL was strong, indicating health professions students were negotiating the demands of SRLE despite course modality challenges. Finally, the mediation model with self-efficacy helped explain the relationship between each social, cognitive, and teaching presence and FSRL.

Despite previous studies emphasis on CoI as a valid framework for investigating planned online learning [23–25] the confidence in framework stability in SRLEs was uncertain, particularly in health professions education. Our findings illuminate the relationship between CoI types and self-efficacy [11] signifying the robustness of these findings. Specifically, health professions students who experience positive online experiences [46–48], likely through faculty interactions and efforts, may have lessened the issues and barriers associated with the course modality changes. In line with the previous research, such interactions may have also helped students’ participation and attitude [15, 16].

These results have both theoretical and practical implications. This study tested the theoretical framework of CoI and its three presence types establishing it as a relevant and stable framework for investigating sustained remote learning environments. Considering the likelihood of continued pandemic-related lockdowns and the need for institutions to prepare for other modality-altering threats [20], the study minimizes the presumption of importance of self-selection for online modalities and emphasizes the role of self-efficacy.

Attitudes about learning environments have changed in recent years. Prior to the COVID-19 pandemic, most health professions education took place in face-to-face settings [69] and transitioning online presented numerous challenges such as lack of training and institutional infrastructure to provide support [70–72]. Moreover, medical and health profession student attitudes toward online learning were stable over the prior 10 years with positive prior experiences correlated with satisfaction and increased knowledge [73]. Our results may inform practitioners in facilitating SRLEs because our data were collected during the stage of COVID-19 in which long term impacts were beginning to be felt [74]. SRLEs are likely to continue [4], and institutions have a demonstrated need to manage course modality changes [20].

### Recommendations

This study demonstrates that student attitudes about non-traditional learning environments is dependent upon CoI presence and self-efficacy, both of which may be influenced through institutional efforts led by program directors and faculty. Health professions program directors may view the found relationship between CoI presence and self-efficacy considering the entire curriculum, evaluating efforts to drive presence and supporting student self-efficacy as a variety of course modalities are offered by the program.

Faculty may take advantage of the findings of this study by devising strategies to address student attitudes and self-efficacy in SRLEs. Simple and practical strategies to increase social, cognitive, and teaching presence, such as inviting personal stories and engaging in a variety of communication platforms, may positively impact student attitudes. Furthermore, identifying course designs which aid in self-efficacy improvement (task difficulty moderation, student autonomy, etc.) and exposing students to social modeling and mastery experiences to increase self-efficacy [39, 75] may, based on the findings of this study, improve student attitudes in SRLEs.

In this later phases of the COVID-19 pandemic, there has been accelerated attention and emphasis on the quality of the learning environments and student outcomes across health professions programs [76]. The pandemic has clearly highlighted the increasing role that technology will play along the continuum of knowledge acquisition and for clinical skill development. These changes have helped to improve the educational process by providing an alternative method of connecting student and faculty. This trend will only accelerate as pressures increase to develop and deliver optimal remote learning environments. Findings from this study within the current CoI framework suggest there are many elements to support a sustained remote health professions educational environment including the perspectives of the student learner, practices that surround that learning experience, and virtual spaces in which it occurs.

Limitations of this study are that the sample may not be representative of all students enrolled in health professions programs. We recognize the vast diversity in student populations that future research should attend to, including more specific student populations as the curriculum and resources in these programs vary, and randomized sampling to limit selection bias [77]. Nonetheless, this study provides valuable insight into self-efficacy and student attitudes in emerging learning environments. Although this study was focused on the relationship between latent constructs in an emerging learning environment, more studies with larger datasets can be used to replicate and confirm findings. Given the nature of the observational data, no causal inference should be made about the relationships. In addition, this study examined CoI in the SRLE context of the United States, and although CoI has been studied in international contexts for online environments, the international SRLE context has not been fully explored.

Finally, this study delimited the exploration of race, ethnicity, and gender because the focus of this analysis was to assess the value of the CoI framework within a SRLE context. Because self-efficacy is known to be affected by race, ethnicity, and gender [43, 44, 78], future researchers may explore demographic effects, or their moderating role, in a CoI/SRLE model.

## Conclusion

The findings of this study are timely and relevant to current health professions program initiatives related to sustained remote learning. Our findings suggest that teaching and learning concerns at the rapid transition online phase of the pandemic may have diminished over time as the sustained remote learning environment becomes more stable. Results showed direct and indirect effects for teaching presence and self-efficacy and social presence and self-efficacy on students’ attitudes toward sustained remote learning, which have both theoretical and practical impacts for sustained remote teaching and learning. Support focused on broadening knowledge and skills of teaching and learning in SRLE may be beneficial.

## Data Availability

The datasets generated during and/or analyzed during the current study are available from the corresponding author on reasonable request.
